# Weifuchun alters tongue flora and decreases serum trefoil factor I levels in gastric intestinal metaplasia: A CONSORT-compliant article

**DOI:** 10.1097/MD.0000000000031407

**Published:** 2022-11-11

**Authors:** Zhaolai Hua, Rui Shen, Bin Lu, Meifeng Li, Ping Zhou, Juan Wu, Wei Dong, Qihai Zhou, Junfeng Zhang

**Affiliations:** a Institute of Tumor Prevention and Control, People’s Hospital of Yangzhong City, Yangzhong, China; b Guangxi Key Laboratory of Rare and Endangered Animal Ecology, Guangxi Normal University, Guilin, China; c School of Medicine & Holistic Integrative Medicine, Nanjing University of Chinese Medical, Nanjing, China; d Department of Oncology, People’s Hospital of Yangzhong City, Yangzhong, China.

**Keywords:** gastric intestinal metaplasia, tongue flora, trefoil factor I, Weifuchun

## Abstract

To explore the molecular mechanisms of Weifuchun in the treatment of gastric intestinal metaplasia (GIM), we designed a preclinical pilot study to examine potential markers of disease progression based on alterations in the tongue flora.

**Methods:**

Total 27 patients with GIM were treated with Weifuchun for 4 weeks and 26 volunteers as controls. Tongue coating bacteria were profiled using 16S rDNA high-throughput sequencing. Serum pepsinogen I and II levels were detected using the latex immunoturbidimetric assay. The levels of serum trefoil factor I was detected by ELISA. Microplate-based quantification was used to detect serum total bile acid (TBA).

**Results:**

After treatment, the relative abundance of 4 dominant tongue coating genera (*Granulicatella*, *Gemella*, *Lachnoanaerobaculum*, and *Neisseria*) increased significantly wheras *Alloprevotella*, *[Eubacterium] nodatum group*, *Prevotell*, and *Ruminococcaceae UCG-014* decreased (*P* < .05). The results showed that *Alloprevotella* and 3 rare tongue coating genera (*Lautropia*, *Treponema* 2, and *Aliihoeflea*) might be potential markers or target flora for the treatment of GIM. Kyoto encyclopedia of genes and genomes (KEGG) function prediction analysis showed that Weifuchun may regulate bile secretion and folate biosynthesis in patients with GIM. The level of serum trefoil factor I decreased significantly in response to Weifuchun treatment, which was consistent with the decrease in folate biosynthesis predicted by KEGG.

**Conclusion:**

Weifuchun may restore the balance of tongue flora by decreasing the levels of serum trefoil factor I, thereby providing a new way to measuring the underlying effectiveness and potential mechanisms of action of this traditional Chinese medicinal compound in the treatment of GIM.

## 1. Introduction

Gastric intestinal metaplasia (GIM) is characterized by chronic inflammation of the gastric mucosa and atrophy of the proper glands, where normal gastric mucosal epithelial cells are replaced with Paneth cells, goblet cells, and absorption cells. GIM dramatically increases the risk of gastric cancer (GC) and is a recognized pathological marker of precancerous gastric lesions.^[1]^ Studies have found that the development of GIM is affected by a large number of factors, such as *Helicobacter pylori* (Hp) infection, heredity, environmental milieu, diet, gastric flora (*Firmicutes*, enterohepatic *Helicobacter* species, *Bacteroidetes*).^[2]^ Early intervention in GIM can reduce the incidence of stomach cancer, although there is no consensus on a treatment approach.^[3]^

Traditional Chinese medicine (TCM) has unique advantages for the treatment of chronic gastritis. TCM considers that GIM results from impaired spleen and stomach function, which can be induced by improper diet, irregular eating, overeating spicy and high-fat foods, smoking and drinking, emotional disorders, and other stressors. TCM treatment methods include strengthening the spleen nourishing the Yin, promoting Qi, and activating the blood to clear heat and disperse stagnation.^[4]^ Weifuchun is a famous Chinese patent drug composed of *Radix Ginseng Rubra* (red ginseng), *Rabdosia amethystoides*, and fried *Fructus aurantii.* Many natural product studies have discovered a great number of chemical components in Weifuchun, such as naringin, various ginsenosides (Re, Rg1, Rb1, Rf, Rb2, Rg2, Rd, and 20 [R]-Rg3), oridonin, epinodosinol, and others.^[5,6]^ According to the TCM theory, Weifuchun can invigorate Spleen-Qi, promote blood circulation, and enhance detoxification. Many clinical investigations have demonstrated its curative effects on stomach diseases such as chronic atrophic gastritis, GIM, and GC postoperative recovery.^[7]^ Recently, Weifuchun has been listed as a potential treatment strategy for precancerous gastric mucosa lesions in China.^[8]^ The underlying mechanism of the therapeutic effects of Weifuchun is related to the downregulation of C-erBb-2 and upregulation of Rb expression,^[9]^ inhibition of Hp proliferation and inflammatory factors such as interleukin (IL)-4, IL-8, and transforming growth factor-β.^[10]^ Tian et al found that Weifuchun may ameliorate gastric mucosal atrophy and intestinal metaplasia in rats by activating homeobox protein NKX6.3, downregulating type homeobox gene Cdx2, upregulating SRY-box containing gene 2, and inhibiting bone morphogenetic protein 4 expression.^[11]^ Li et al conducted a biomolecular network analysis and isentified tumor necrosis factor, IL-6, and tumor-related signaling pathways (JAK/STAT3 and PI3K/ATK) among its potential targets in treating chronic atrophic gastritis.^[12]^ However, the molecular mechanisms underlying the therapeutic effects of Weifuchun on gastric precancerous lesions remain unclear.

The mouth joins to the stomach via the esophagus, so stomach disorders such as esophageal reflux can affect the oral environment and tongue. According to TCM theory, the coating of the tongue reflects the pathophysiological state of the Spleen-Stomach (generally referring to digestive functions), especially in gastric diseases. The flora is a crucial component of the tongue -coating along with desquamated epithelial cells. Many studies demonstrated that tongue flora can provide potential biomarkers for the diagnosis of certain diseases, measurement of disease progression and prognosis, and efficacy of different treatments.^[13,14]^ Jiang et al found that a white coating (generally representing the cold syndrome) and a yellow coating (generally representing the hot syndrome) reflected different bacterial compositions and commensal relationships in patients with chronic gastritis.^[15]^ Ye et al found that Bacillus was a potential marker of the yellow tongue coating in patients with chronic erosive gastritis, as it disappeared after 2 courses of treatment with Banxia Xiexin decoction. The color of tongue coating also changes from yellow to white.^[16]^ Our previous findings showed that the bitter pungent dispersion purgation method could effectively treat patients with upper gastrointestinal precancerous lesions and significantly change their tongue coating flora.^[17]^ Cui et al found that the relative abundance of *Campylobacter concisus* in tongue coating increased substantially with the progression of gastritis, chronic atrophic gastritis, and intestinal metaplasia.^[18]^ Wu et al revealed that an increase in the relative abundance of *Streptococcus* in the tongue coating was significantly related to GC risk.^[19]^ Xu et al found that changes in the tongue flora may be related to inflammation and metabolism but not to lifestyle changes, suggesting that the tongue -coating flora may provide noninvasive biomarkers for GC diagnosis.^[20]^ Therefore, we designed a preclinal study to prove that treatment of GIM with Weifuchun could lead to alterations in tongue flora and provide novel insights into the biological mechanisms of effective therapy.

## 2. Patients and methods

### 2.1. Patients

Twenty-seven patients with GIM diagnosed by painless gastroscopy at the People’s Hospital of Yangzhong City from April 2017 to June 2018 were recruited for Weifuchun treatment. Twenty-six volunteers were enrolled in the same period and matched to GIM patients by age, gender, and body mass index, according to the diagnostic criteria of the “Consensus Opinions on Chinese Early Gastric Cancer Screening and Endoscopy Diagnosis and Treatment” (2014, Changsha, China). This study was approved by the Clinical Ethics Committee of the People’s Hospital of Yangzhong City (No. PHYC201605) and all patients provided informed consent. Exclusion criteria were: a history of taking drugs in the last 4 weeks, such as proton pump inhibitors, probiotics, antibiotics, and non-steroidal anti-inflammatory drugs; a history of esophageal or gastric mucosal surgery; suffering from gastric mucosal intraepithelial neoplasia, gastric cancer or other digestive severe system diseases; a history of heart, lung, liver, or kidney dysfunction or mental disorders; pregnant or breastfeeding women; and an allergic constitution, particularly a history of allergy to Weifuchun.

### 2.2. Drug intervention

Weifuchun tablets (40 tablets per box, Hangzhou Huqing Yutang Pharmaceutical Co., Ltd., National Medicine Standard: Z20040003, Lot. 1806648) were distributed to the patients once every 2 weeks. The patients took 4 tablets at a time, 3 times a day, as instructed. Weekly follow-ups were conducted to guide patients to take medication on time and record the degree of upper gastrointestinal symptoms, including halitosis, heartburn, acid reflux, stomachaches, and appetite. After 4 weeks of treatment and 1 week of drug withdrawal, pathological reexamination was performed using painless gastroscopy.

### 2.2. Clinical evaluation

The effects of treatment were determined by gastroscopic pathology based on criteria established in “Precancerous lesions of gastric cancer: Foundation and clinic,”^[21]^ and the criteria used in the evaluation were as follows: Cure: intestinal metaplasia disappears; Significantly effective: inflammation is reduced by 1 grade, and intestinal metaplasia is reduced by 1 grade; Effective: inflammation is reduced by 1 grade or intestinal metaplasia is reduced by 1 grade; Ineffective: there was no significant improvement or deterioration in inflammation and intestinal metaplasia. The total effective rate equals the sum of the cure, apparent, and effectiveness rates.

The 8 evaluation indicators for the cumulative symptom score were halitosis, heartburn, acid reflux, nausea, hiccups, belching, loss of appetite, and gastric pain. The cumulative score was based on the following criteria: no symptoms = 0; light = 3; medium = 5; and heavy = 7. After treatment, a reduction of 2/3 in the cumulative score was considered a cure. A reduction of 1/2 was a marked effect, a reduction of 1/4 was effective, and a reduction of <1/4 was inconclusive, with the cumulative score remaining unchanged.

### 2.3. Immunoassay

During gastroscopy, a rapid chemical reaction test strip (Guangzhou Besiqi Reagent Co., Ltd.) was used to detect Hp infection in the stomach. Latex immunoturbidimetric assay was used to detect the levels of serum pepsinogen-I (PG-I; pepsinogen I assay kit, Cat. No. G17001, Lot. No. 19041006, Beijing Leadman Biochemistry Co., Ltd.) and pepsinogen II (PG–II; Pepsinogen II assay kit, Cat. No. 11701, Lot. No. 19051410, Beijing Leadman Biochemistry Co., Ltd.). Enzyme-linked immunosorbent assay (ELISA) was conducted to detect the levels of serum Trefoil factor 1 (TFF1; Human pS2/ Trefoil factor 1 ELISA Kit, Cat. No. ARG81530, Arigobio). Serum total bile acid (TBA) level was measured using a microplate-based quantification assay (Total Bile Acid Kit, No. E003-2-1, Nanjing Jiancheng Bioengineering Institute, China). The assays were performed according to the manufacturer’ instructions.

Briefly, the serum and kits were equilibrated at room temperature (22°C~23°C) until the serum completely melted. Serum TBA levels were tested using an enzymatic cycling method.^[22]^ 180 μL reagent 1 (R1) and 3 μL standard samples/serum samples were incubated for 5 minutes at 37 °C; 60 μL reagent 2 (R2) was incubated for 1 min at 37°C, and the first absorbance (A1) was measured at 405 nm using a microplate reader. The second absorbance (A2) was measured at 405 nm after incubating for 3 minutes at 37°C. The TBA concentration was calculated using the difference method (∆A = A2–A1). The level of TFF1 was measured by ELISA.^[23]^ Total 100 μL of standards/serum samples was added to wells and incubated for 90 minutes at 37°C. After carefully removing the supernatant, 100 μL of avidin labeled antibody was added and incubated for 60 minutes at 37°C. Then, the plate was washed 3 times, and 100 μL of streptavidin labeled horseradish peroxidase was added and incubated for 30 minutes at 37°C. After the plate was washed 5 times, 90 μL substrate (tetramethylbenzidine) was added to each well and incubated for 20 minutes at 37°C in the dark. 100 μL of stop solution halted the reaction, and the OD was immediately measured at 450 nm. Finally, serum TFF1 concentration was calculated using a standard curve.

### 2.4. Analysis of tongue coating flora

Prior to gastroscopy, patients were instructed to gargle 2 to 3 times with sterile normal saline. The tongue coating was brushed 5 to 10 times with a sterile toothbrush, the brushed deposits were suspended in the sterile saline, with the precipitate centrifuged at 1800×*g* for 10 minutes, and the washed deposits were stored at −80°C.

Tongue coating flora was detected by Shanghai BIOZERON Co., Ltd. as previously reported.^[20]^ Briefly, total DNA of the tongue coating was extracted using 16S rDNA universal primers (338F-ACTCCTACGGGAGGCAGCAG; 806R-GGACTACHVGGGTWTCTAAT) for PCR amplification. The PCR products were used to prepare the Hiseq PE library and high-throughput sequencing was performed. QIIME (version 1.17) was used to optimize the sequencing data. The operational taxonomic unit (OTU) was established using the UPARSE35 (version 7.1 http://drive5.com/uparse/) software with 97% similarity. Visual Genomics (Release 1, Shanghai Infinity Biotechnology Co., Ltd.) was used for data normalization and bioinformatic analysis. Alpha diversity analysis was performed prior to data normalization. Beta diversity analysis, community structure analysis, linear discriminant analysis (LDA), and Kyoto encyclopedia of genes and genomes (KEGG) function prediction were performed after data normalization.

### 2.5. Statistical analysis

Statistical analyze were performed using SPSS 21.0. Normal-distribution of data was presented as mean ± standard deviation (SD) and analyzed using Student’s *t* test. Qualitative data was analyzed using the chi-squared test. The non-normal distribution of data is presented as median (Q25, Q75), and a non-parametric test was used. All tests for significance were two-sided, and *P* values < .05 were statistically significant.

## 3. Results

### 3.1. General clinical information

After 4 weeks of Weifuchun treatment, among the 27 GIM patients, gastroscopy pathology showed that 19 patients were treated effectively, symptom score showed that 18 patients benefited from Weifuchun treatment (8 cured cases, 4 significantly effective cases, and 6 effective cases) (Table S1, Supplemental Digital Content, http://links.lww.com/MD/H779). Altogether, 11 patients benefitted from Weifuchun treatment by both gastroscopy pathology and symptom score, 9 patients benefitted from Weifuchun treatment by gastroscopy pathology, and 7 patients benefitted from Weifuchun treatment by cumulative symptom score. The common characteristics of the 27 patients with GIM and 26 controls are shown in Table 1. There were no statistically significant differences between the groups (*P* > .05).

**Table 1:** The common characteristic of the present population.

	Controls (n = 26)	GIM patients (n = 27)	χ^2^/*t* (*P*)
Gender (male/female)	12/14	12/15	0.016 (.901)^*^
Age	63.7 ± 2.5	63.0 ± 3.6	0.767 (.446)^†^
BMI	23.5 ± 3.0	24.6 ± 3.8	1.097 (.278)^†^
Hp-infection (positive/negative)	5/21	11/16	2.908 (.088)^*^

BMI = body mass index, GIM = gastric intestinal metaplasia.

* Chi square test.

† Student *t* test.

### 3.2. Effect of weifuchun on the diversity of tongue coating flora

**Figure 1.**
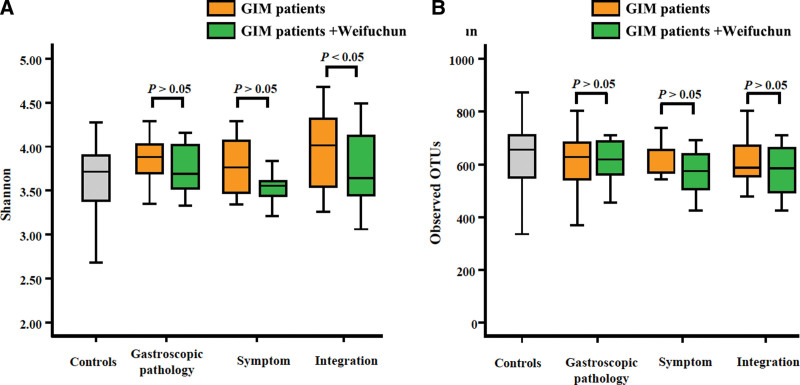
The alpha diversity analysis of tongue coating flora. (a) The diversity differences based on Shannon index. (b) The diversity differences based on observed OTUs. OTU = operational taxonomic unit.

The Kruskal–Wallis *H* test showed no significant differences in the bacterial diversity of the tongue coating among the 3 groups (*P* > .05, Fig. 1). The Mann–Whitney *U* test showed that the Shannon index of the tongue coating flora was significantly reduced in patients with GIM treated with Weifuchun (*P* < .05, Fig. 1a). However, there were no significant differences between the observed OTUs (*P* > .05, Fig. 1b). These results suggest that Weifuchun reduces the diversity of tongue coating flora in patients with GIM.

To further explore the impact of Hp infection on the diversity of tongue coating flora, we found that the Simpson index of tongue coating flora of Hp-negative GIM patients was significantly lower than that of the Hp-negative controls (*P* < .05, Figure S1 Supplemental Digital Content, http://links.lww.com/MD/H780).

### 3.3. Effect of weifuchun on the structure of tongue coating flora

**Figure 2.**
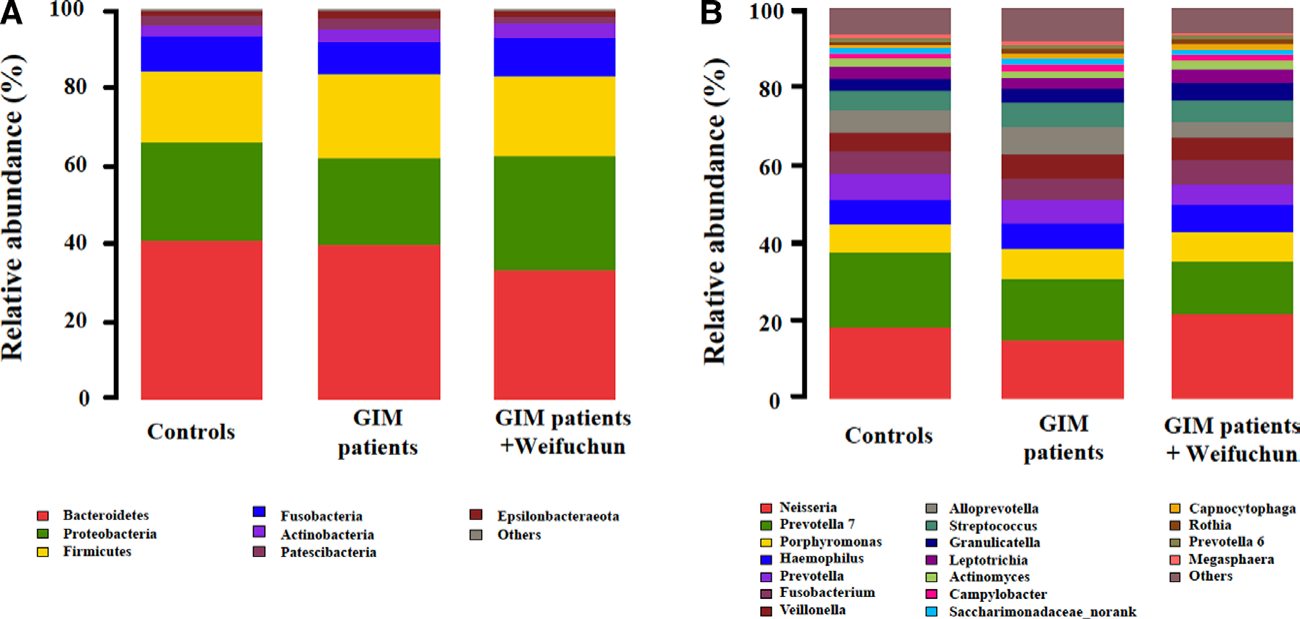
The structure of the tongue coating microbiota of GIM patients treated with Weifuchun. (a) Composition of the dominant bacterial phyla in the tongue coating. (b) Composition of the dominant bacterial genera in the tongue coating. GIM = gastric intestinal metaplasia.

After data normalization, 995 OTUs were obtained from the tongue coating of all samples, distributed among 28 phyla, 49 classes, 120 orders, 231 families, and 532 genera. The relative abundances of bacteria at the phylum and genus levels are shown in Figure 2. The dominant bacteria phyla (relative abundance >1%) from high to low are *Bacteroidetes*, *Proteobacteria*, *Firmicutes*, *Fusobacteria*, *Actinobacteria*, *Patescibacteria*, and *Epsilonbacteraeota* (Fig. 2a). The top 10 dominant bacteria were *Neisseria*, *Prevotella-7*, *Porphyromonas*, *Haemophilus*, *Prevotella*, *Fusobacterium*, *Veillonella*, *Alloprevotella*, *Streptococcus*, and *Granulicatella* (Fig. 2b).

Kruskal–Wallis *H* test and Mann–Whitney *U* test were conducted to screen the dominant phyla and genera of the tongue coating flora. At the phylum level, the relative abundances of *Bacteroidetes* and *Spirochaetes* were significantly altered among the 3 groups (*P* < .05) and were significantly reduced following Weifuchun treatment (*P* < .05, Table 2). Moreover, Weifuchun treatment dramatically increased *Proteobacteria* and decreased *Tenericutes* in the tongue coating of patients with GIM (*P* < .05).

**Table 2:** The analysis of dominant phyla of tongue coating flora (Median [Q25, Q75] %).

Phylum	Controls (n = 26)	GIM patients (n = 27)		*H* (*P*)	*Z* (*P*)^*^	*Z* (*P*)^†^	*Z* (*P*)^‡^
		Before treatment	After treatment				
*Actinobacteria*	2.78 (1.54, 3.49)	2.59 (1.80, 3.50)	2.47 (1.84, 5.94)	0.454 (.797)	0.231 (.817)	0.667 (.505)	0.424 (.672)
*Bacteroidetes*	40.00 (34.68, 48.31)	40.81 (34.87, 43.73)	31.78 (25.25, 39.44)	9.177 (.010)	0.000 (1.000)	2.776 (.006)	2.448 (.014)
*Epsilonbacteraeota*	1.38 (0.98, 1.61)	1.23 (0.83, 2.29)	1.02 (0.65, 1.75)	1.491 (.474)	0.036 (.972)	1.112 (.266)	0.995 (.320)
*Firmicutes*	16.98 (13.46, 25.37)	18.63 (11.36, 28.02)	20.28 (14.32, 26.22)	0.498 (.780)	0.587 (.557)	0.623 (.533)	0.130 (.897)
*Fusobacteria*	8.38 (5.77, 11.52)	7.72 (5.51, 10.17)	7.32 (5.82, 13.72)	0.540 (.763)	0.569 (.569)	0.231 (.817)	0.657 (.511)
*Patescibacteria*	1.58 (0.54, 3.28)	2.67 (1.00, 3.41)	1.34 (1.02, 2.66)	1.693 (.429)	0.818 (.413)	0.249 (.803)	1.341 (.180)
*Proteobacteria*	25.54 (15.40, 36.76)	21.74 (14.98, 30.04)	30.60 (18.80, 41.09)	4.211 (.122)	0.818 (.413)	1.139 (.255)	2.067 (.039)
*Spirochaetes*	0.07 (0.03, 0.10)	0.09 (0.04, 0.22)	0.03 (0.01, 0.07)	8.038 (.018)	1.335 (.182)	1.638 (.101)	2.735 (.006)
*Tenericutes*	0.01 (0.00, 0.02)	0.02 (0.00, 0.02)	0.00 (0.00, 0.01)	4.261 (.119)	0.935 (.350)	0.972 (.331)	2.121 (.034)

GIM = gastric intestinal metaplasia.

* GIM patients versus Controls.

† GIM patients with Weifuchun treatment vs Controls.

‡ GIM patients versus GIM patients with Weifuchun treatment.

At the genus level, the relative abundances of *Granulicatella*, *Alloprevotella*, and the *[Eubacterium] nodatum group* were significantly different (*P* < .05). However, no differences were observed between the patients with GIM and controls. Patients with GIM benefitting from Weifuchun treatment showed 3 significantly increased genera (*Granulicatella*, *Gemella*, and *Lachnoanaerobaculum*) in the tongue coating (*P* < .05) and 3 significantly decreased genera (*Alloprevotella*, *[Eubacterium] nodatum group*, and *Prevotella*) (*P* < .05). Furthermore, increases in *Granulicatella* and *Neisseria* and decreases in *Alloprevotella* and *Ruminococcaceae UCG-014* were observed in the tongue coating of patients with GIM following Weifuchun treatment (*P* < .05, Table 3).

**Table 3:** The different dominant genera of tongue coating flora among the 3 groups (Median [Q25, Q75] %).

菌属	Controls (n = 26)	GIM patients (n = 27)		*H* (*P*)	*Z* (*P*)^*^	*Z* (*P*)^†^	*Z* (*P*)^‡^
		Before treatment	After treatment				
*Granulicatella*	2.18 (1.64, 3.94)	2.42 (1.49, 4.16)	4.28 (2.82, 5.88)	11.536 (.003)	0.383 (.702)	3.220 (.001)	2.586 (.010)
*Neisseria*	19.87 (9.27, 26.83)	13.58 (10.26, 19.06)	23.63 (14.52, 29.94)	5.470 (.065)	0.907 (.364)	1.085 (.278)	2.483 (.013)
*Alloprevotella*	5.37 (3.70, 7.65)	6.48 (2.81, 11.01)	2.95 (1.62, 5.09)	8.827 (.012)	1.085 (.278)	2.562 (.010)	2.431 (.015)
*Ruminococcaceae UCG-014*	0.23 (0.10, 0.42)	0.32 (0.12, 0.61)	0.12 (0.05, 0.29)	4.660 (.097)	0.792 (.428)	1.379 (.168)	2.094 (.036)
*[Eubacterium] nodatum group*	0.71 (0.44, 1.27)	0.55 (0.37, 0.91)	0.35 (0.28, 0.65)	6.686 (.035)	1.032 (.302)	2.456 (.014)	1.704 (.088)
*Gemella*	0.36 (0.21, 0.50)	0.28 (0.14, 0.74)	0.57 (0.28, 1.26)	4.103 (.129)	0.311 (.756)	1.975 (.048)	1.523 (.128)
*Prevotella*	6.57 (5.00, 8.19)	6.21 (3.00, 7.91)	4.27 (2.04, 6.98)	3.972 (.137)	0.685 (.493)	2.082 (.037)	1.081 (.280)
*Lachnoanaerobaculum*	0.20 (0.14, 0.28)	0.25 (0.15, 0.43)	0.28 (0.15, 0.47)	4.759 (.093)	1.379 (.168)	2.127 (.033)	0.839 (.401)

GIM = gastric intestinal metaplasia.

* GIM patients versus Controls.

† GIM patients with Weifuchun treatment vs Controls.

‡ GIM patients versus GIM patients with Weifuchun treatment.

### 3.4. Analysis of tongue coating bacteria as potential biomarkers for weifuchun treatment

**Figure 3.**
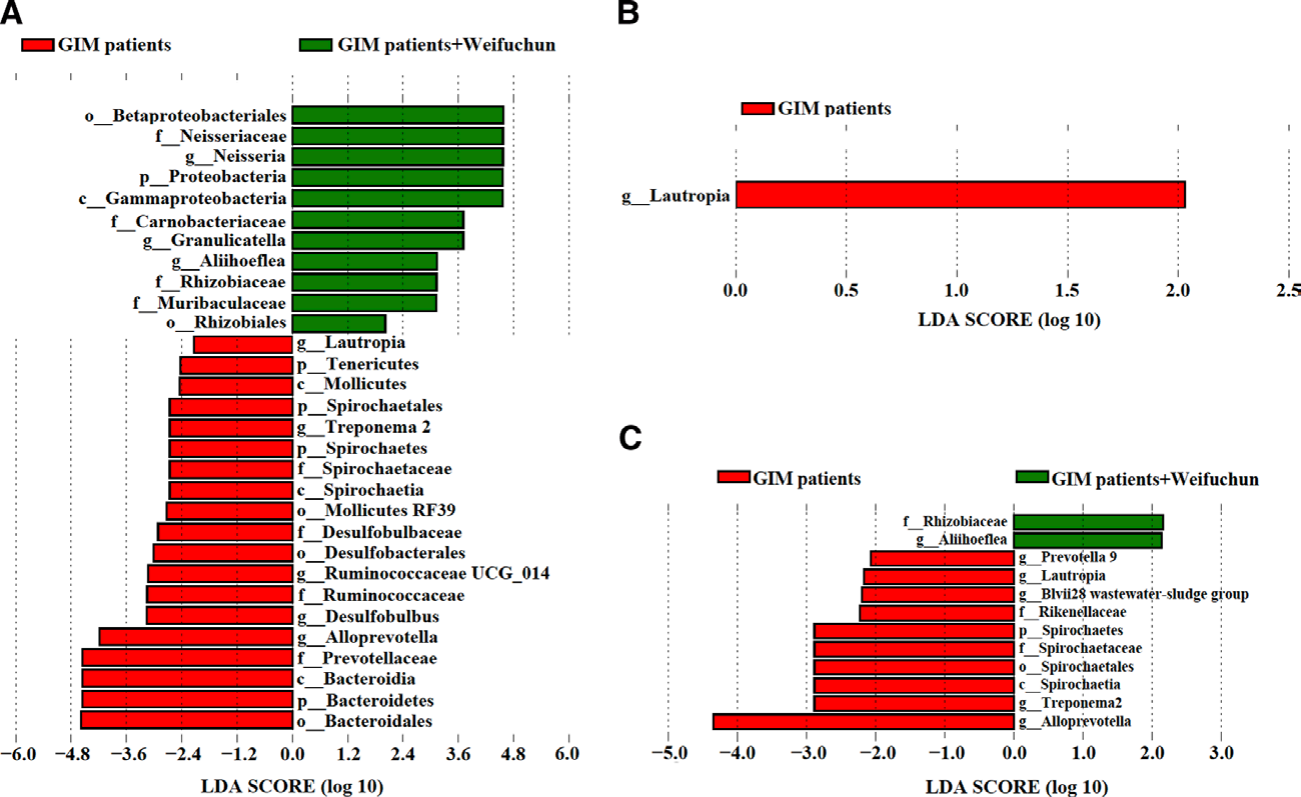
Linear discriminant analysis (LDA) of tongue coating microbiota in GIM patients before and after Weifuchun treatment. (a) The 27 GIM patients benefited from Weifuchun treatment by the integrated evaluation. (b) The 19 GIM patients benefited from Weifuchun treatment based on the assessment of gastroscopic pathology. (c) The 18 GIM patients benefited from Weifuchun treatment based on TCM symptom scores. GIM = gastric intestinal metaplasia, TCM = traditional Chinese medicine.

We then sought to examine potential marker flora relevant to measuring the effects of Weifuchun treatment, LEfSe analysis was performed on the tongue flora among the 3 groups, although no significant differences were detected (data not shown). Further stratified analysis showed that 11 tongue coating bacterial taxa (1 phylum, 2 class, 2 orders, 4 families, and 3 genera) were enriched in the GIM patients benefitting from Weifuchun treatment (Fig. 3a). Only *Lautropia* was enriched in the 14 patients with GIM based on gastroscopy pathology (Fig. 3b), and family *Rhizobiaceae* and genus *Aliihoeflea* were enriched in the 13 patients with GIM benefitting from Weifuchun treatment according to symptom scores (Fig. 3c). Taken together, the decreased tongue coating of *Lautropia* was the collective genus most relevant to the Weifuchun treatment.

**Figure 4.**
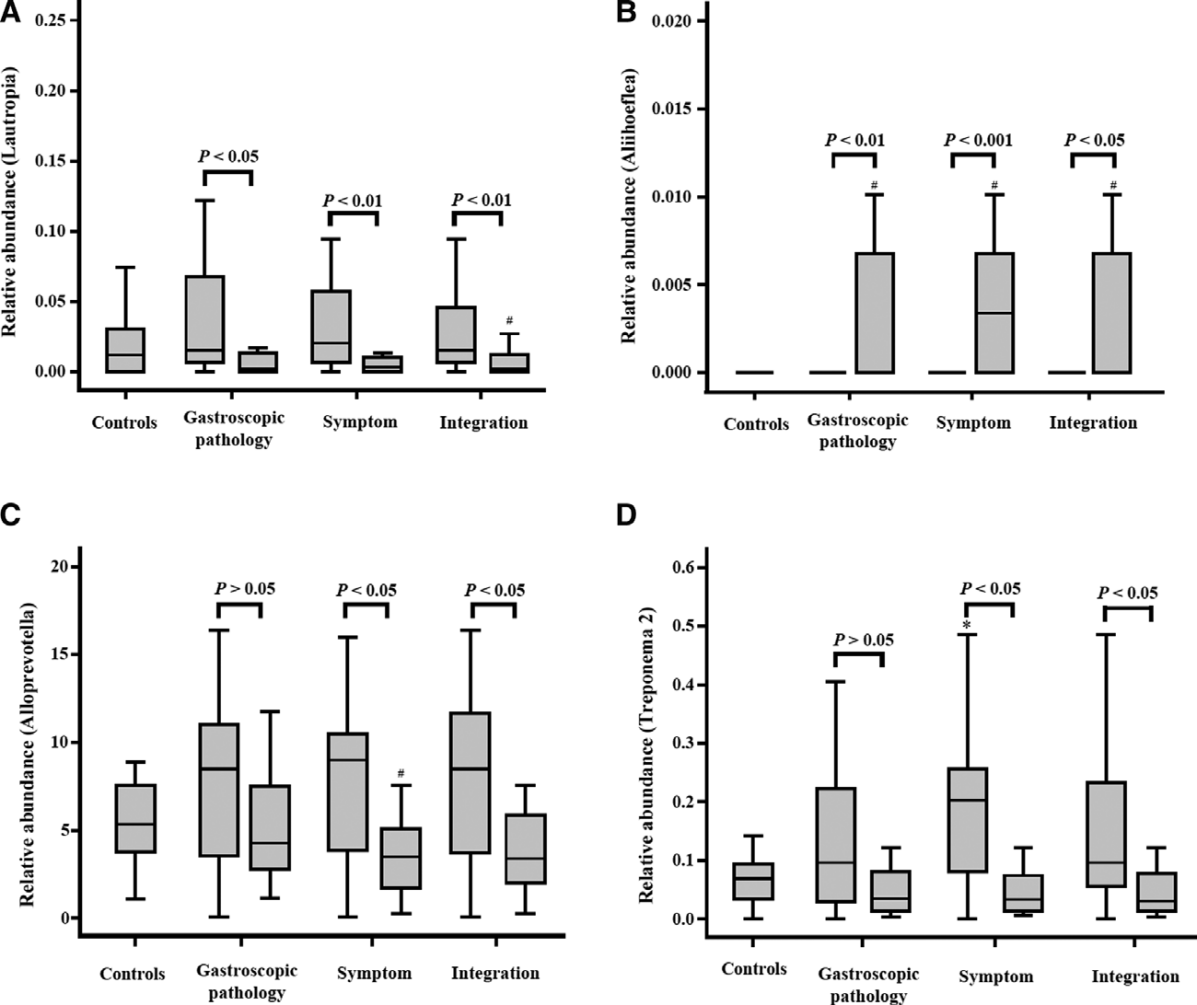
Different analysis of the 4 genera along with the different evaluation methods of Weifuchun treatment. Compared with the controls, *means the significant difference in GIM patients (*P* < .05), # means the significant difference in GIM patients with Weifuchun treatment (*P* < .05). GIM = gastric intestinal metaplasia.

Furthermore, the Mann–Whitney *U* test revealed that treatment with Weifuchun significantly increased the abundance of *Aliihoeflea* but reduced the abundance of *Lautropia*, *Alloprevotella*, and *Treponema 2* in the tongue coating of patients with GIM (Fig. 4). Therefore, these 4 genera might be potential marker bacteria for evaluating the effectiveness of Weifuchun when used in the treatment of GIM.

### 3.5. Predictive functions affected by weifuchun treatment

**Figure 5.**
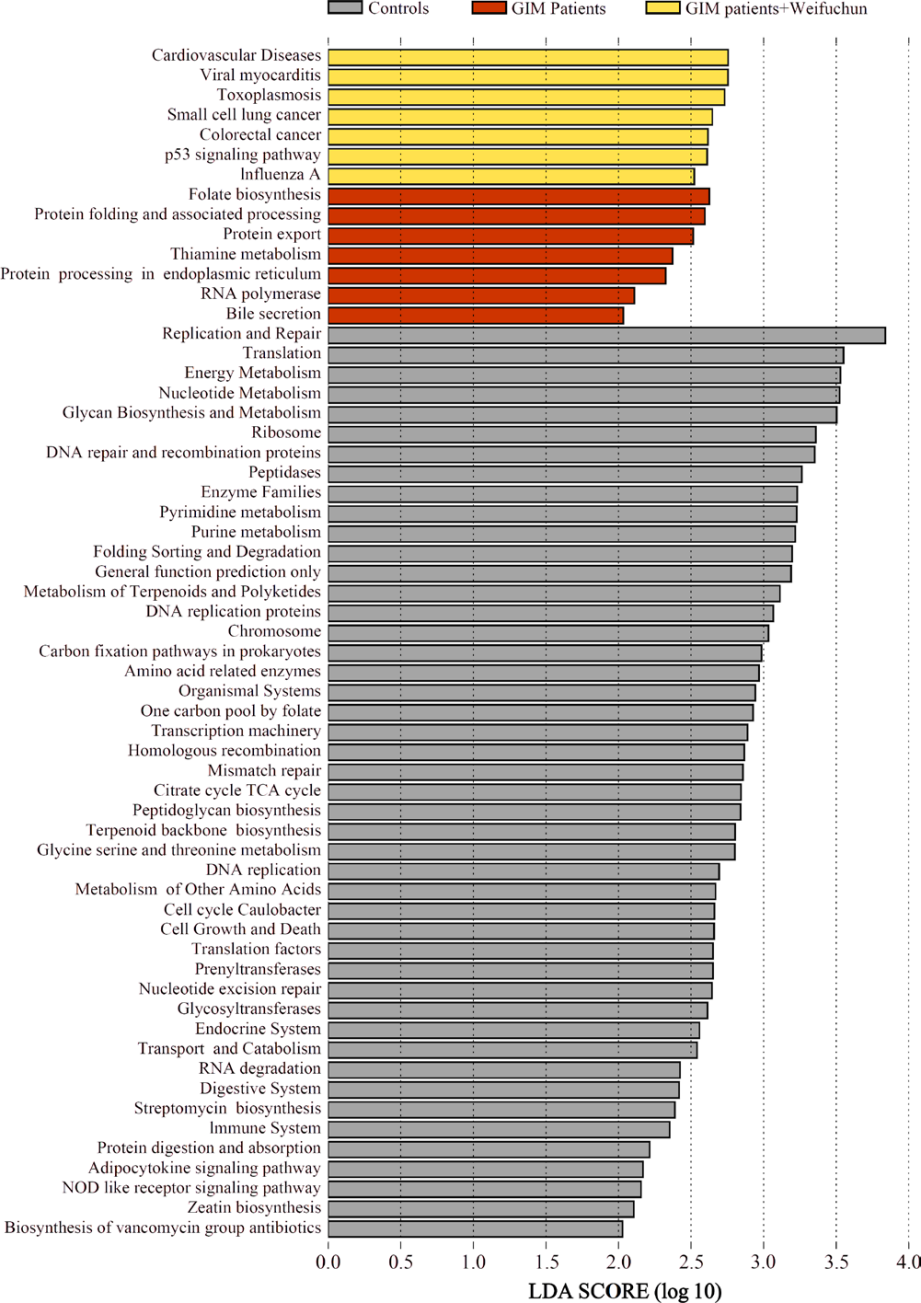
LDA of KEGG predictive functions of tongue coating flora among the controls, the GIM patients, and GIM patients with Weifuchun treatment. GIM = gastric intestinal metaplasia, LDA = linear discriminant analysis, KEGG = Kyoto encyclopedia of genes and genomes.

**Figure 6.**
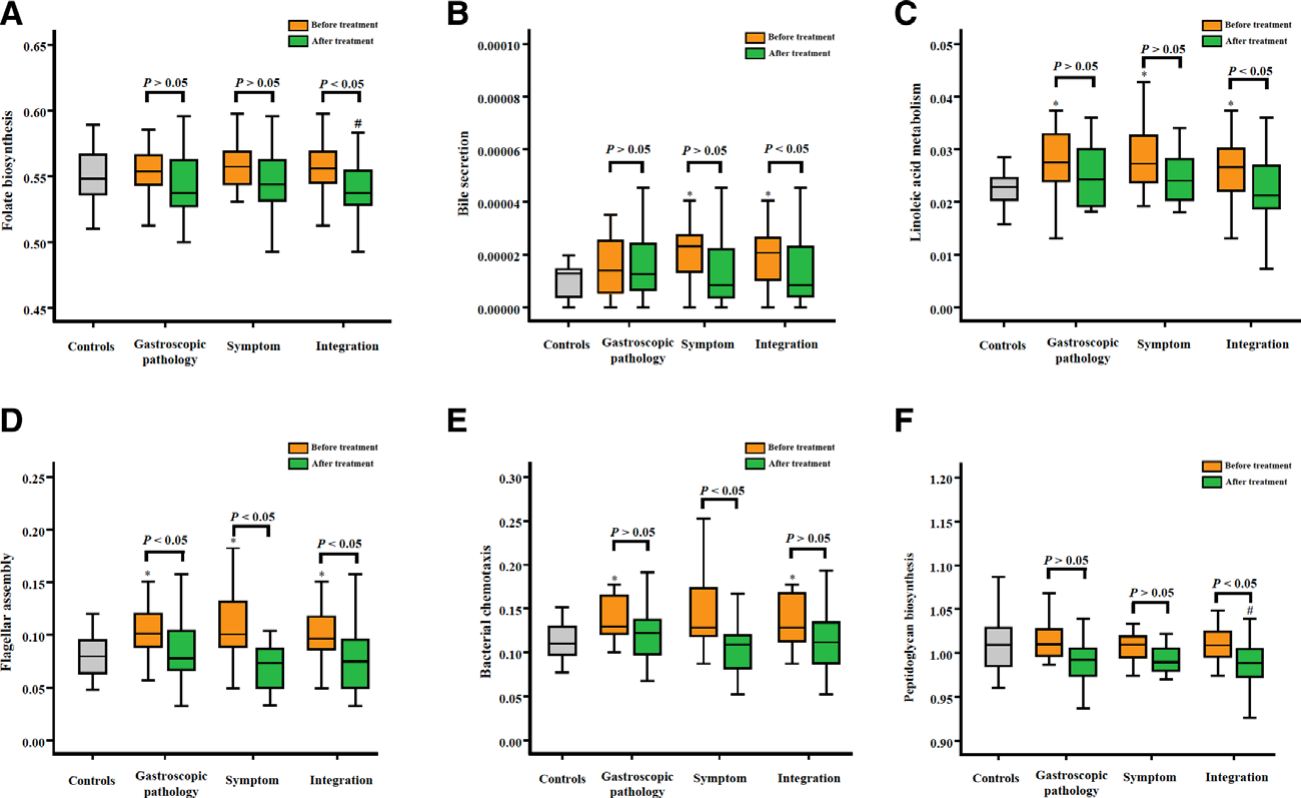
Different analysis of 6 predictive functions based on tongue coating flora. Compared with the controls, *means the significant difference in the GIM patients (*P* < .05), # means the significant difference in the GIM patients with Weifuchun treatment (*P* < .05). GIM = gastric intestinal metaplasia.

To explore the underlying mechanism of the effects of Weifuchun treatment on GIM, LDA was performed based on the KEGG predictive function of the tongue coating flora. The results showed 60 predictive functions relevant to the Weifuchun treatment (Fig. 5). Mann–Whitney *U* analysis showed that 7 predictive functions were significantly reversed by Weifuchun treatment, including 3 metabolism-related functions (folate biosynthesis, bile secretion, and linoleic acid metabolism) and 3 bacteria-related functions (flagellar assembly, bacterial chemotaxis, and peptidoglycan biosynthesis) (Fig. 6). These findings suggest that Weifuchun affect bile secretion and folate biosynthesis in patients with GIM by inhibiting flagellar assembly, peptidoglycan biosynthesis, and bacterial chemotaxis.

### 3.6. Network analysis of core bacteria in tongue coating

**Figure 7.**
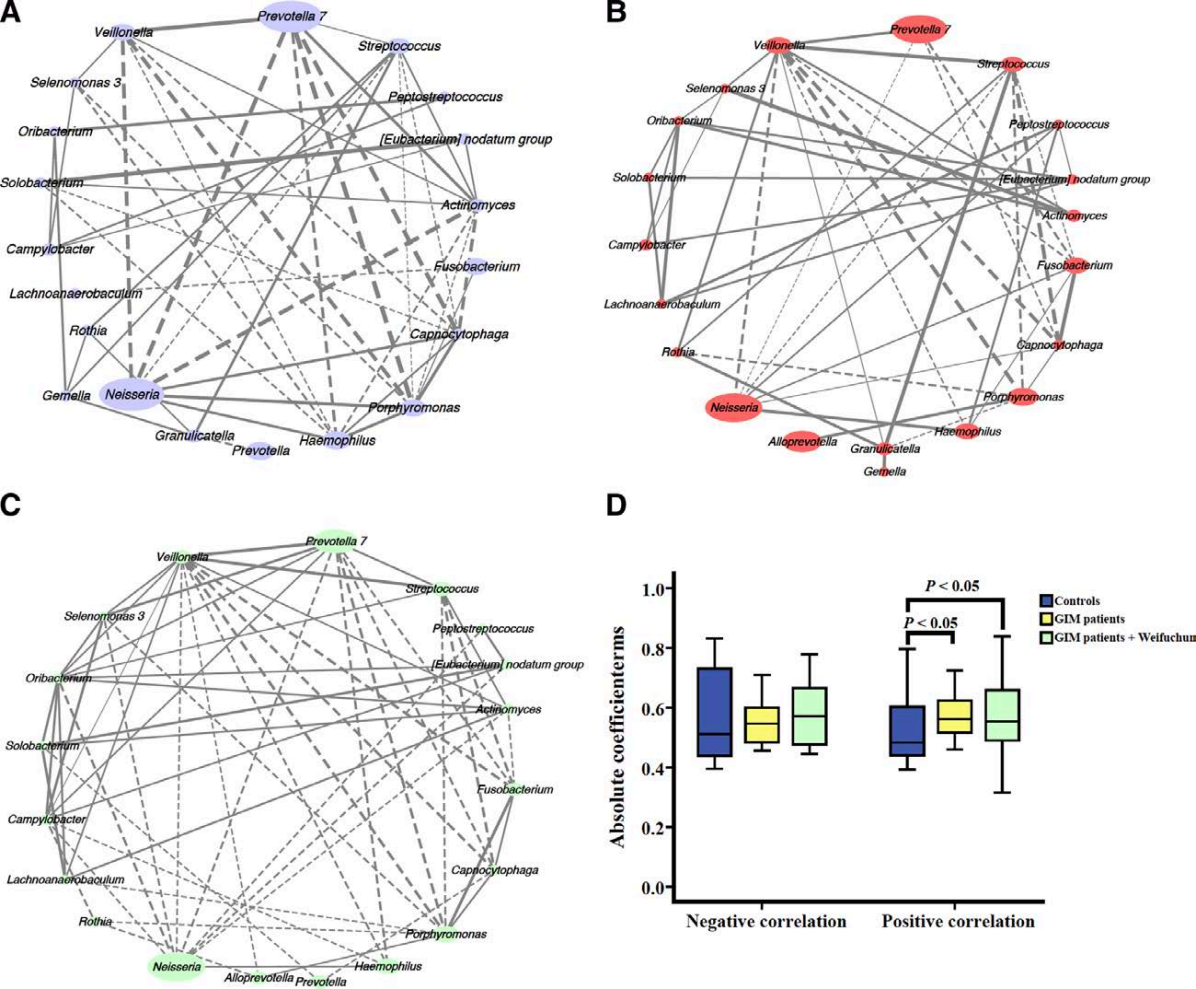
Network analysis of the core bacteria in the tongue coating. (a) The controls. (b) The GIM patients. (c) The GIM patients with Weifuchun treatment. (d) The different analysis of the correlation coefficients among the 3 groups. The solid line means positive correlation, the dotted line means negative correlation, and the size of the node means relative abundance. GIM = gastric intestinal metaplasia.

Twenty core tongue coating genera with an average relative abundance >0.1% and 100% distribution across the total population were selected to construct a symbiotic network by Spearman’s correlation analysis (Fig. 7). The controls had a symbiotic network, including 21 negative and 28 positive correlations (Fig. 7a). patients with GIM before Weifuchun treatment had a symbiotic network, including 14 negative correlations and 33 positive correlations (Fig. 7b), and patients with GIM treated with Weifuchun had a symbiotic network consisting of 29 negative correlations and 31 positive correlations (Fig. 7c). Compared with the controls, the positive correlation coefficient of dominant tongue coating flora genera in GIM patients was significantly increased, but the negative correlation coefficient did not change significantly (Fig. 7d), suggesting that abnormal proliferation occurred in tongue coating patients with GIM. Notably Weifuchun treatment in GIM patients restored the positive *Streptococcus* - *Prevotell*a *7* correlation, while the positive *Neisseria* - *Peptostreptococcus* correlation became negative, which may be related to the significantly increased abundance of *Neisseria*, an important typical oral bacterium (Table 3). These results suggest that Weifuchun treatment partially restored the balance of tongue coating flora.

### 3.7. Verification of the predictive function of tongue coating flora

**Figure 8.**
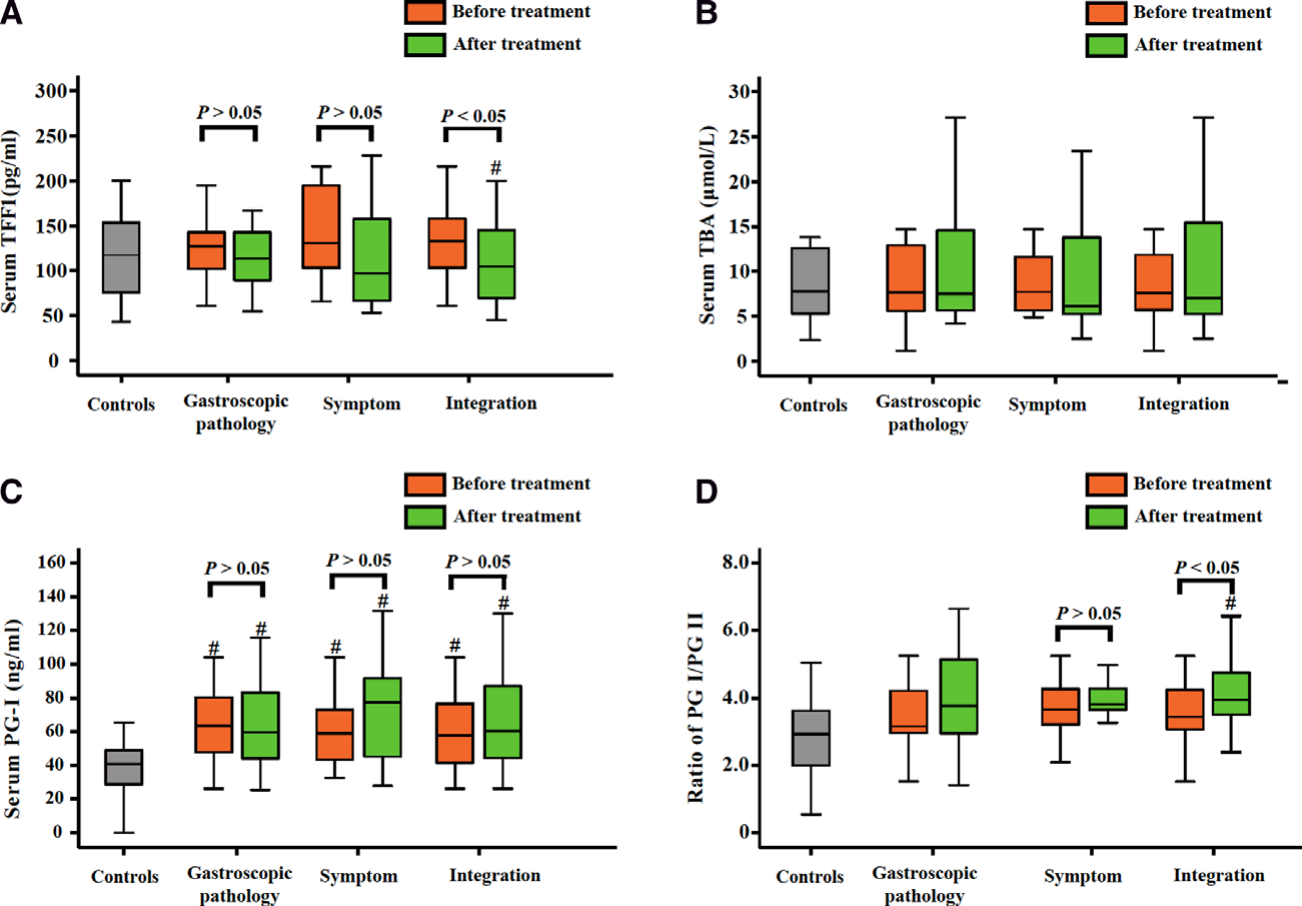
The verification of the predictive function of tongue coating flora. Comparison with the controls, # means the significant differences (*P* < .05).

Serum pepsinogen (PG) is commonly used as an early diagnostic marker of GC. PG, secreted by the gastric mucosa, is divided into PG-I and PG-II and enters the blood through the capillaries of the gastric mucosa.^[24]^ Therefore, serum PG levels are a key marker of the morphology and function of the gastric mucosa. Decreased gastric basal mucosa leads to decreased PG-I levels, whereas PG-II levels remain unchanged. Serum PG-I levels and the PG-I/PG-II ratio can therefore reflect the degree of gastric mucosal atrophy.^[25]^ Here, we analyzed the serum levels of TBA, TFF-1, PG-I, and PG-II, based on the results of the predictive function of the tongue coating flora in GIM patients with Weifuchun treatment (Fig. 8). The results showed that serum TFF1 levels were significantly reduced after Weifuchun treatment (*P* < .05, Fig. 8a), consistent with the decrease inpredictive folic acid biosynthesis function. However, the serum TBA levels did not change significantly (*P* > .05, Fig. 8b). Compared with the controls, GIM patients had significantly increased levels of serum PG-I (*P* < .05, Fig. 8c) but not serum PG-II (*P* > .05). After 4 weeks of Weifuchun treatment, the ratio of serum PG-II/PG-I in patients with GIM was significantly higher than that in controls (*P* < .05, Fig. 8d), which was similar to a previous report.^[26]^

## 4. Discussion

GC is the fifth most common malignant tumor worldwide, and its incidence and mortality rank third and second among malignant tumors in China, making it a serious challenge to Chinese public health.^[27,28]^ Intestinal GC usually progresses from normal gastric mucosa to non-atrophic gastritis, atrophic gastritis, intestinal metaplasia, and eventually cancer (Correa’s cascade).^[29]^ As a part of this process, GIM is recognized as a typical gastric precancerous lesion. Therefore, early intervention from GIM, could decrease the risk and incidence of GC. Weifuchun is an effective Chinese patent medicine for the treatment of GIM.

Bacterial dysbiosis is associated with several digestive system diseases and cancers. Change in microbial biodiversity are regarded as typical features of ecological dysbiosis.^[30]^ Wang et al found that the microbial diversity of the gastric mucosa in early gastric cancer was lower than that seen in advanced gastric cancer.^[31]^ Cui et al found that the tongue coating flora of patients with gastritis has a low species richness, but the diversity (Shannon index) is significantly higher than that of controls.^[17]^ However, Xu et al failed to find significant differences in the richness and diversity of tongue coating flora in patients.^[32]^ The present results did not show significant differences in the diversity of tongue coating flora between GIM patients and controls, but Weifuchun treatment greatly reduced the diversity of the tongue coating flora in GIM patients, which suggests that Weifuchun may have an effect on regulating microbial balance.

Hp is a well-known type I carcinogen associated with GC. It is considered one of the main causes of GC development, so its elimination could significantly reduce the risk of GC.^[33]^ Indeed, the American Gastroenterological Association strongly recommends testing and treatment of patients with GIM for Hp eradication.^[34]^ Park et al found that the abundance of gastric *Rhizobiales* in Hp-negative GIM patients was significantly higher than that in Hp-negative chronic superficial gastritis and GC patients.^[35]^ Zhao et al^[36]^ found that Hp infection, especially cytotoxin associated gene A-positive, could substantially alter the diversity and composition of the gastric mucosal flora and tongue -coating flora in patients with chronic gastritis. The prediction function based on gastric flora showed that Hp infection upregulated the lipopolysaccharide biosynthesis function, which might weaken the protective effect of oral flora against pathogens. This study also observed a decrease in the diversity of tongue coating flora in patients with Hp-negative GIM. These results suggest that Hp infection plays an essential role in the oral flora.

Regarding the dominant genera, Weifuchun treatment significantly increased the abundance of *Gemella*, *Granulicatella*, *Lachnoanaerobaculum*, and *Neisseria*, but significantly decreased the abundance of *Alloprevotella*, *[Eubacterium] nodatum group*, *Prevotell*, and *Ruminococcaceae UCG-014*. Among them, *Granulicatella* (gram-positive facultative anaerobic cocci) and *Gemella* (gram-positive cocci) were the normal flora in the human oral cavity and gastrointestinal tract.^[37,38]^ Our previous study^[19]^ found that tongue coating *Alloprevotell* was related to increased GC risk, whereas *Neisseria* and *Lachnoanaerobaculum* were related to decreased GC risk. Weifuchun treatment increased the abundance of tongue *Lachnoanaerobaculum* and *Neisseria* and reduced the abundance of *Alloprevotella*. These results suggest that Weifuchun treatment can decrease the risk of GC, at least based on its influence on tongue coating flora, which provides an indirect readout of the effectiveness of Weifuchun in preventing and treating gastric cancer.

LDA showed that *Alloprevotella* and 3 non-dominant genera (*Lautropia*, *Treponema 2*, and *Aliihoeflea*) might be potential markers of Weifuchun treatment in GIM. *Lautropia* is a gram-negative facultative anaerobic bacterium. Saliva *Lautropia* was positively related to *Streptococcus*, a pro-inflammatory bacterium, in patients with Barrett’s esophagus.^[39]^ In hepatitis B patients, saliva *Lautropia* was significantly increased, but it was not observed in hepatitis B patients with cirrhosis, liver cancer patients, or healthy controls, suggesting that *Lautropia* may be an early signal of hepatitis B virus infection.^[40]^ Our previous study observed decreased tongue *Lautropia* in patients with GC,^[20]^ which was consistent with the results of saliva *Lautropia* in patients with oral cancer^[41]^ and esophageal squamous cell carcinoma.^[42]^ These results suggest that *Lautropia* could be a potential marker flora of precancerous lesions in the oral cavity, inaddition to its presence in the tongue coating. Here, we found that Weifuchun treatment significantly reduced the abundance of tongue coating *Lautropia* in patients with GIM, which may correlate with an improvement in gastric mucosal precancerous lesions. So far, there have been few studies on *Aliihoeflea*. Zhang et al^[43]^ showed that *Aliihoeflea* could be used as a marker for dextran sodium sulfate-induced enteritis intestinal flora disorders. Gong et al^[44]^ found a protective effect of the anti-aging Chinese patent drug Formula Kang Shuai Lao Pian, which could also reduce the abundance of intestinal *Aliihoeflea* in obese mice induced by a high-fat diet. However, our results showed that Weifuchun treatment increased the abundance of tongue coating *Aliihoeflea* in patients with GIM. These differences might be due to the different locations of the tongue coating and the intestine.

The interaction between bacteria is a critical factor in maintaining microbiome homeostasis of the. Wang et al^[31]^ found that GC patients demonstrated less complexity of the bacteria network than chronic gastritis patients, which was consistent with the results reported by Liu et al^[45]^ However, Ferreira et al^[46]^ found that the microbiome imbalance index of the tissue surrounding gastric tumors was higher and the network was more complex than that of normal gastric mucosa. These findings indicate that the loss of certain bacterial interactions in GC leads to the disruption of homeostasis in the microbiota of the stomach. The symbiotic network analysis in our study suggested that compared with controls, Weifuchun treatment could restore the synergistic relationship between *Streptococcus* and *Prevotell*a *7* in the tongue coating of patients with GIM. This restoration might be related to the significant increase in the abundance of *Neisseria*, which was observed to correlate with a decreased risk of GC in a previous study.^[19]^ These results indicate that Weifuchun treatment is related to the restoration of the normal balance of tongue flora.

Based on the KEGG predictive function of the tongue coating flora, Weifuchun treatment might function by inhibiting flagellar assembly, peptidoglycan biosynthesis, and bacterial chemotaxis while also affecting bile secretion and folate biosynthesis in GIM patients. Bacterial flagella and peptidoglycan are important components for inducing an inflammatory response, and flagellar assembly and bacterial chemotaxis play significant roles in cancer-related inflammation.^[47]^ Peptidoglycan is the main component of the bacterial cytoderm and the target of many antibiotics. Peptidoglycan biosynthesis is closely related to cytoderm growth and renewal of the cytoderm.^[48]^ The pathways participating in peptidoglycan biosynthesis may regulate inflammation in tumors by enhancing intestinal cell permeability.^[49]^ Grivennikov et al^[50]^ found that peptidoglycan biosynthesis may play a role in GC development. Zhang et al^[51]^ found that the bacterial motility function (bacterial chemotaxis and flagellar assembly) of the oral flora was significantly enriched in oral cancer patients, which was similar to our results showing that Weifuchun treatment inhibited bacterial chemotaxis and flagellar assembly function.

TFF1, a small molecule peptide, is highly expressed in the human stomach. TFF1 is involved in maintaining the structure and function of the gastric mucosa, protecting it against damage, inhibiting gastric acid secretion, and stimulating mucosal cell proliferation.^[52,53]^ Shi et al^[54]^ found that miR-632 could improve tube formation and recruitment of endothelial cells by negatively regulating the expression of TFF1 in GC cells, and the upregulation of TFF1 in para-cancerous tissues might be associated with its function in tumor suppression. High expression of serum TFF1 is related to an increased risk of gastric, lung, pancreatic and breast cancers.^[55]^ Huang et al^[56]^ found that GC patients had higher levels of serum TFF1 than controls and patients with chronic non-atrophic gastritis. Shimura et al^[57]^ found that GC patients had significantly higher urine levels of TFF1, disintegrin, and metalloproteinase domain-containing protein 12 than healthy controls, indicating that urine TFF1 and metalloproteinase domain-containing protein 12 could be used as early diagnostic markers of GC. Here, we observed that Weifuchun treatment significantly reduced serum TFF1 levels in patients with GIM, which verified the downregulated predictive function of folate biosynthesis.

However there still have several limitations such as: it was just a single arm trial, and here did not choose any positive control drugs; the dietary characteristics possibly changed after one-month treatment, which might also affect the flora in tongue coating; here did not analyze the tongue coating flora in the patients who didn’t benefit from Weifuchun treatment.

## 5. Conclusion

Weifuchun, an effective Chinese patent drug, is widely used to treat many kinds of stomach disorders, especially to prevent and ameliorate precancerous lesions of gastric cancer. In this pre-clinical study, we found that alterations in the tongue coating flora were correlated with the effectiveness of Weifuchun treatment in patients with GIM, which has established a foundation for a large-scale multicenter study to analyze its therapeutic effects by regulating the balance of the digestive tract flora in treating GIM.

## Acknowledgments

We are grateful to the students who participated in the collection and arrangement of clinical data, Miss Chunjie Xiang, Mr. Shuo Xu, Miss Jiaqian Huang, Miss Qiyi Li, and Miss Zhouyi Wang. This work wsa supported by the National Natural Science Foundation of China (81473458, 81973737), the Chinese Medicine Science and Technology Program of Jiangsu Province (Yb2021023), and Guangxi Key Laboratory of Rare and Endangered Animal Ecology.

## Author contributions

Zhaolai Hua and Rui Shen have contributed equally to this work. Junfeng Zhang and Qihai Zhou conceived of and designed the study. Zhaolai Hua designed the clinical scheme. Bin Lu and Ping Zhou were responsible for the clinical consultation and doctor-patient communication. Rui Shen, Meifeng Li, Juan Wu and Wei Dong acquired and analyzed the clinical data. Rui Shen, Zhaolai Hua and Junfeng Zhang completed the graphic of the data display and drafted the manuscript. All authors have read and approved the final manuscript.**Conceptualization:** Zhaolai Hua, Qihai Zhou, Junfeng Zhang.

**Data curation:** Zhaolai Hua, Rui Shen, Meifeng Li, Ping Zhou, Juan Wu, Wei Dong, Qihai Zhou, Junfeng Zhang.

**Formal analysis:** Rui Shen, Meifeng Li, Juan Wu, Wei Dong, Qihai Zhou, Junfeng Zhang.

**Funding acquisition:** Qihai Zhou, Junfeng Zhang.

**Investigation:** Bin Lu, Ping Zhou, Junfeng Zhang.

**Methodology:** Zhaolai Hua.

**Project administration:** Qihai Zhou, Junfeng Zhang.

**Resources:** Junfeng Zhang.

**Supervision:** Qihai Zhou, Junfeng Zhang.

**Writing – original draft:** Zhaolai Hua, Rui Shen, Junfeng Zhang.

**Writing – review & editing:** Qihai Zhou, Junfeng Zhang.

## Supplementary Material



## References

[1] Shao LM Li PW Ye J. Risk of gastric cancer among patients with gastric intestinal metaplasia. Int J Cancer. 2018;143:1671–7. 29707766 10.1002/ijc.31571

[2] Jencks DS Adam JD Borum ML. Overview of current concepts in gastric intestinal metaplasia and gastric cancer. Gastroenterol Hepatol. 2018;14:92–101. PMC5866308 29606921

[3] Trieu JA Bilal M Saraireh H. Update on the diagnosis and management of gastric intestinal metaplasia in the USA. Dig Dis Sci. 2019;64:1079–88. 30771043 10.1007/s10620-019-05526-5

[4] Zhang SS Tang XD Huang HP. Expert consensus on diagnosis and treatment of traditional Chinese medicine of chronic gastritis (2017). China J Tradit Chin Med Pharmacy. 2017;32:3060–4.

[5] Zhang HX Zhang LF Zhang L. Simultaneous determination of 11 constituents in Weifuchun tablets by HPLC-MS/MS. Chin J Clin Pharmacol. 2018;34:1470–4. (In Chinese)

[6] Jin YR Tian TT Ma YH. Simultaneous determination of ginsenoside Rb1, naringin, ginsenoside Rb2 and oridonin in rat plasma by LC-MS/MS and its application to a pharmacokinetic study after oral administration of Weifuchun tablet. J Chromatogr B Analyt Technol Biomed Life Sci. 2015;1000:112–9. 10.1016/j.jchromb.2015.06.027 26222904

[7] Gu ZJ Ling JH Cong J. A review of therapeutic effects and the pharmacological molecular mechanisms of Chinese medicine Weifuchun in treating precancerous gastric conditions. Integr Cancer Ther. 3215;19:2020. 10.1177/1534735420953215 PMC7466872 32865036

[8] National Clinical Research Center for Digestive Disease (Shanghai), National Early Gastrointestinal-Center Prevention & Treatment Center Alliance, Helicobacter Pylori Group, Chinese Society of Gastroenterology, Chinese Medical Association, Chinese Health Management Association, Digestive endoscopy Committee of Endoscopists Branch of Chinese Medical Association and Cancer endoscopy Committee of China Anti-Cancer Association. Chinese consensus on management of gastric epithelial precancerous conditions and lesions (2020). Chin J Dig. 2020;40:731–41. (In Chinese)

[9] Shu M Jiang Z Wang YY. Clinical efficacy of Weifuchun combined with retinoic acid on treatment of patients with PLGC and effect on expressions of gene Rb and C-erbB-2. Biomed Res. 2017;28:606–9.

[10] Zhang YQ Ke Y Guan Y. Clinical application and research progress of Weifuchun pills in chronic atrophic gastritis and precancerous lesions of gastric cancer. Shanghai Med Pharma J. 2018;39:43–6. (In Chinese)

[11] Tian FL Yang XJ Chen WQ. Effects of Yiwei Xiaoyu Granule on intestinal metaplasia of gastric mucosa in rats with atrophic gastritis. J Basic Chin Med. 2020;26:1080–3. (In Chinese)

[12] Li P Li Y Li KX. Mechanism of Weifuchun tablets in treating chronic atrophic gastritis based on biomolecular network. World J Integr Tradit West Med. 2020;15:48–53. (In Chinese)

[13] Sun SP Wei HM Zhu RL. Biology of the tongue coating and its value in disease diagnosis. Complement Med Res. 2018;25:191–7. 28957816 10.1159/000479024

[14] Wu J Qian J Shi LY. Application value of tongue coating microflora in integration of traditional Chinese medicine and modern medicine. TMR Theory Hypothesis. 2019;2:229–40.

[15] Jiang B Liang XJ Chen Y. Integrating next-generation sequencing and traditional tongue diagnosis to determine tongue coating microbiome. Sci Rep. 2012;2:936. 23226834 10.1038/srep00936 PMC3515809

[16] Ye J Cai XT Yang J. Bacillus as a potential diagnostic marker for yellow tongue coating. Sci Rep. 2016XXX;6;2496. 10.1038/srep32496 PMC5006162 27578261

[17] Sun X Xu J Hua ZL. Effects of pungent dispersion bitter purgation method on bacterial flora of tongue coating in 24 patients with precancerous lesions of upper gastrointestinal. China J Tradit Chin Med Pharma. 2018;33:3322–6. (In Chinese)

[18] Cui JX Cui HF Yang MR. Tongue coating microbiome as a potential biomarker for gastritis including precancerous cascade. Protein Cell. 2019;10:496–509. 30478535 10.1007/s13238-018-0596-6 PMC6588651

[19] Wu J Xu S Xiang CJ. Tongue coating microbiota community and risk effect on gastric cancer. J Cancer. 2018;9:4039–48. 30410609 10.7150/jca.25280 PMC6218773

[20] Xu S Xiang CJ Wu J.Tongue coating Bacteria as a potential stable biomarker for gastric cancer independent of lifestyle Digestive Diseases and Sciences. 2021;66:2964–2980. 33044677 10.1007/s10620-020-06637-0

[21] Lao SX. Precancerous Lesions of Gastric Cancer: Foundation and Clinic. Guangzhou: Guangdong People’s Publishing House; 2002.

[22] Tian G Ding M Xu B. A novel electrochemical biosensor for ultrasensitive detection of serum total bile acids based on enzymatic reaction combined with the double oxidation circular amplification strategy. Biosens Bioelectron. 2018;118:31–5. 30055417 10.1016/j.bios.2018.07.030

[23] Yi J Ren L Li D. Trefoil factor 1 (TFF1) is a potential prognostic biomarker with functional significance in breast cancers. Biomed Pharmacother. 2020;124:109827. 31986408 10.1016/j.biopha.2020.109827

[24] Cai QC Zhu CP Yuan Y. Development and validation of a prediction rule for estimating gastric cancer risk in the Chinese high-risk population: a nationwide multicenter study. Gut. 2019;68:1576–87. 30926654 10.1136/gutjnl-2018-317556 PMC6709770

[25] Tong Y Wang H Zhao Y. Diagnostic value of serum pepsinogen levels for screening gastric cancer and atrophic gastritis in asymptomatic individuals: a cross-sectional study. Front Oncol. 2021;11:652574. 34504781 10.3389/fonc.2021.652574 PMC8421685

[26] Wang J Yang B Li L. Effect of Banxia Xiexin decoction combined with Weifuchun in the treatment of serum epidermal growth factor, serum pepsinogen and gastrin expression in patients with chronic atrophic gastritis. J Liaoning Univ Tradit Chin Med. 2019;21:154–7.

[27] Bray F Ferlay J Soerjomataram I. Global cancer statistics 2018: GLOBOCAN estimates of incidence and mortality worldwide for 36 cancers in 185 countries. CA Cancer J Clin. 2018;68:394–424. 30207593 10.3322/caac.21492

[28] Feng RM Zong YN Cao SM. Current cancer situation in China: good or bad news from the 2018 Global Cancer Statistics? Cancer Commun (London, England). 2019;39:22. 10.1186/s40880-019-0368-6 PMC6487510 31030667

[29] Waldum H Fossmark R. Gastritis, gastric polyps and gastric cancer. Int J Mol Sci . 2021;22:6548. 34207192 10.3390/ijms22126548 PMC8234857

[30] Carding S Verbeke K Vipond DT. Dysbiosis of the gut microbiota in disease. Microb Ecol Health Dis. 2015;26:6191. 10.3402/mehd.v26.26191 PMC4315779 25651997

[31] Wang LL Xin YN Zhou JH. Gastric mucosa-associated microbial signatures of early gastric cancer. Front Microbiol. 2020;11:1548. 32733423 10.3389/fmicb.2020.01548 PMC7358557

[32] Xu J Xiang CJ Zhang C. Microbial biomarkers of common tongue coatings in patients with gastric cancer. Microb Pathog. 2019;127:97–105. 30508628 10.1016/j.micpath.2018.11.051

[33] Pan KF Zhang L Gerhard M. A large randomised controlled intervention trial to prevent gastric cancer by eradication of *Helicobacter pylori* in Linqu County, China: baseline results and factors affecting the eradication. Gut. 2016;65:9–18. 25986943 10.1136/gutjnl-2015-309197

[34] Altayar O Davitkov P Shah SC. AGA technical review on gastric intestinal metaplasia - epidemiology and risk factors. Gastroenterology. 2020;158:732–44. 31816301 10.1053/j.gastro.2019.12.002 PMC7425600

[35] Park CH Lee AR Lee YR. Evaluation of gastric microbiome and metagenomic function in patients with intestinal metaplasia using 16S rRNA gene sequencing. Helicobacter. 2019;24:e12547. 30440093 10.1111/hel.12547 PMC6587566

[36] Zhao YB Gao XF Guo JX. Helicobacter pylori infection alters gastric and tongue coating microbial communities. Helicobacter. 2019;23:e12567. 10.1111/hel.12567 PMC6593728 30734438

[37] Wu LL Tian YJ Li Z. Research progress of Granulicatella species in differentiation and infection correlation. Pract J Med Pharma. 2017;34:1132–3.

[38] Verma D Garg PK Dubey AK. Insights into the human oral microbiome. Arch Microbiol. 2018;200:525–40. 29572583 10.1007/s00203-018-1505-3

[39] Snider EJ Compres G Freedberg DE. Barrett’s esophagus is associated with a distinct oral microbiome. Clin Transl Gastroenterol. 2018;9:135. 29491399 10.1038/s41424-018-0005-8 PMC5862155

[40] Li DX Xi WJ Zhang Z. Oral microbial community analysis of the patients in the progression of liver cancer. Microb Pathog. 2020;149:4479. 10.1016/j.micpath.2020.104479 32920149

[41] Zhao HS Chu M Huang ZW. Variations in oral microbiota associated with oral cancer. Sci Rep. 2017;7:11773. 28924229 10.1038/s41598-017-11779-9 PMC5603520

[42] Chen XD Winckler B Lu M. Oral Microbiota and risk for esophageal squamous Cell carcinoma in a high-risk area of China. PLoS One. 2015;10:e0143603. 26641451 10.1371/journal.pone.0143603 PMC4671675

[43] Zhang F Li Y Wang XL. The impact of Lactobacillus plantarum on the gut microbiota of mice with DSS-induced colitis. Biomed Res Int. 2019;2019:1315 10.1155/2019/3921315 PMC6402223 30915354

[44] Gong SQ Ye TT Wang MX. Traditional Chinese medicine formula Kang Shuai Lao Pian improves obesity, gut dysbiosis, and fecal metabolic disorders in high-fat diet-fed mice. Front Pharmacol. 2020;11:297. 32269525 10.3389/fphar.2020.00297 PMC7109517

[45] Liu XS Li S Liu X. Alterations of gastric mucosal microbiota across different stomach microhabitats in a cohort of 276 patients with gastric cancer. EBio Med. 2019;40:336–48. 10.1016/j.ebiom.2018.12.034 PMC6412016 30584008

[46] Ferreira RM Pereira-Marques J Pinto-Ribeiro I. Gastric microbial community profiling reveals a dysbiotic cancer-associated microbiota. Gut. 2018;67:226–36. 29102920 10.1136/gutjnl-2017-314205 PMC5868293

[47] Al-Hebshi NN Nasher AT Maryoud MY. Inflammatory bacteriome featuring Fusobacterium nucleatum and Pseudomonas aeruginosa identified in association with oral squamous cell carcinoma. Sci Rep. 2017;7:1834. 28500338 10.1038/s41598-017-02079-3 PMC5431832

[48] Wamp S Rutter ZJ Rismondo J. PrkA controls peptidoglycan biosynthesis through the essential phosphorylation of ReoM. Elife. 2020;9:6048. 10.7554/eLife.56048 PMC7286690 32469310

[49] Coker OO Dai ZW Nie YZ. Mucosal microbiome dysbiosis in gastric carcinogenesis. Gut. 2018;67:1024–32. 28765474 10.1136/gutjnl-2017-314281 PMC5969346

[50] Grivennikov SI Wang KP Mucida D. Adenoma-linked barrier defects and microbial products drive IL-23/IL-17-mediated tumour growth. Nature. 2012;491:254–8. 23034650 10.1038/nature11465 PMC3601659

[51] Zhang L Liu Y Zheng HJ. The oral microbiota may have influence on oral cancer. Front Cell Infect Microbiol. 2020;9:476. 32010645 10.3389/fcimb.2019.00476 PMC6974454

[52] Yang Y Lin Z Lin Q. Pathological and therapeutic roles of bioactive peptide trefoil factor 3 in diverse diseases: recent progress and perspective. Cell Death Dis. 2022;13:62. 35039476 10.1038/s41419-022-04504-6 PMC8763889

[53] Yu LL Li RJ Liu W. Protective effects of wheat peptides against ethanol-induced gastric mucosal lesions in rats: vasodilation and anti- inflammation. Nutrients. 2020;12:2355. 10.3390/nu12082355 PMC7469019 32784583

[54] Shi Y Huang XX Chen GB. miR-632 promotes gastric cancer progression by accelerating angiogenesis in a TFF1-dependent manner. BMC Cancer. 2019;19:19. 30612555 10.1186/s12885-018-5247-z PMC6322242

[55] Ishibashi Y Ohtsu H Ikemura M. Serum TFF1 and TFF3 but not TFF2 are higher in women with breast cancer than in women without breast cancer. Sci Rep. 2017;7:4846. 28687783 10.1038/s41598-017-05129-y PMC5501858

[56] Huang ZG Zhang X Lu HN. Serum trefoil factor 3 is a promising non-invasive biomarker for gastric cancer screening: a monocentric cohort study in China. BMC Gastroenterol. 2014;14:74. 24720760 10.1186/1471-230X-14-74 PMC4012276

[57] Shimura T Dayde D Wang H. Novel urinary protein biomarker panel for early diagnosis of gastric cancer. Br J Cancer. 2020;123:1656–64. 32934343 10.1038/s41416-020-01063-5 PMC7686371

