# Supplementary figures and images for: Weifuchun alters tongue flora and decreases serum trefoil factor I levels in gastric intestinal metaplasia: A CONSORT-compliant article
Source: Medicine (Baltimore). 2022 Nov 11;101(45):e31407. doi: 10.1097/MD.0000000000031407 (PMC9666156; doi:10.1097/MD.0000000000031407)

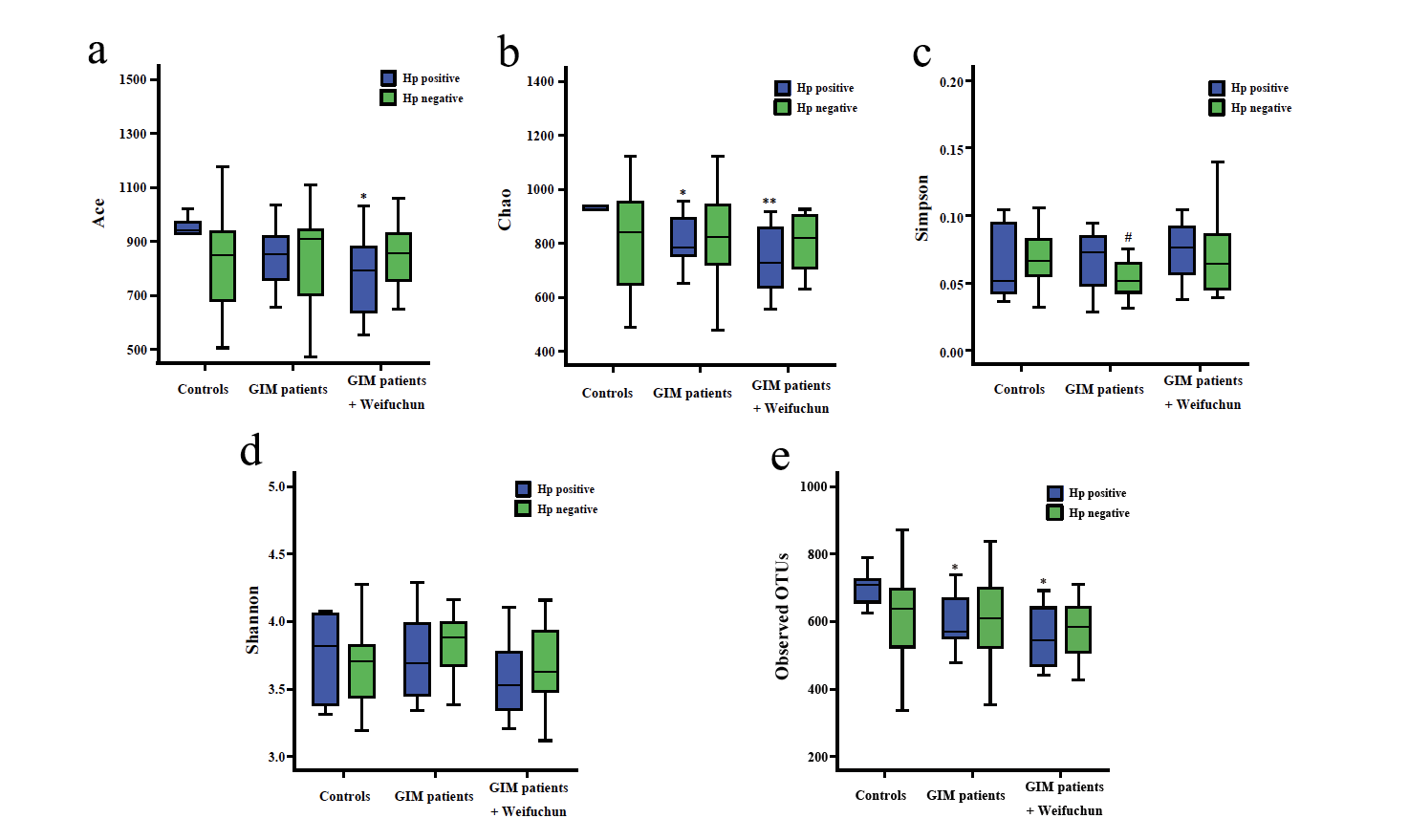

Supplement: Supplementary file 2 [file medi-101-e31407-s002.tif]
